# Unhealthy behaviours associated with uncontrolled hypertension among adults in India- Insights from a national survey

**DOI:** 10.1371/journal.pone.0310099

**Published:** 2025-01-17

**Authors:** Kuppli Sai Sushma, Shubham Kumar, Chaitanya Gujjarlapudi, Vennam Bodhi Srividya, Madhur Verma, N. G. Nagamani, Kishore Yadav Jothula, Nidhi Jaswal, Sonu Goel

**Affiliations:** 1 Department of Community Medicine, Andhra Medical College, Visakhapatnam, India; 2 National Family Health Survey, International Institute for Population Sciences (IIPS), Mumbai, India; 3 Department of Community Medicine, GITAM Institute of Medical Sciences & Research, Visakhapatnam, India; 4 Department of Community & Family Medicine, All India Institute of Medical Sciences Bathinda, Punjab, India; 5 Department of Community & Family Medicine, AIIMS, Bibinagar, Telangana; 6 Arogya World, Spring House, PA, United States of America; 7 Department of Community Medicine and School of Public Health, Post Graduate Institute of Medical Education and Research, Chandigarh, India; West Bengal State University, INDIA

## Abstract

**Background:**

Non-communicable diseases (NCDs) are governed by a cluster of unhealthy behaviours and their determinants, like tobacco and alcohol, unhealthy diet, lack of physical activity, overweight and obesity, pollution (air, water, and soil), and stress. Regulation of these unhealthy behaviours plays a crucial role in blood pressure control among individuals on hypertensive treatment, especially those suffering from uncontrolled hypertension. Hence, the present study aims at identifying the unhealthy behaviours associated with uncontrolled hypertension.

**Materials and methods:**

We did a secondary data analysis of the National Family Health Survey (NFHS) -5 data (2019–2021). Among those taking prescribed medication to lower blood pressure levels, SBP ≥140 mm Hg or DBP ≥90 mm Hg were considered uncontrolled hypertension. The other socio-demographic variables and unhealthy behaviours were used as independent variables for analysis.

**Results:**

The proportion of uncontrolled hypertension was 49·5% (95% CI: 45·5–53·4) and 36·8% (95% CI: 35.8–37.8) among males and females, respectively. Alcohol consumption, clean fuel usage, and high BMI (≥30kg/m^2^) were the behavioural characteristics significantly associated with uncontrolled hypertension among males. In contrast, tobacco usage, alcohol consumption, coverage by Health insurance, presence of Diabetes, heart disease, usage of clean fuel, and high BMI (≥30kg/m^2^) were the behavioural characteristics significantly associated with uncontrolled hypertension among females. Regression results portrayed that higher age groups (45 and above) have found higher odds for men (OR: 7.6, CI: 4.6–12.3) and women (OR: 6.08, CI: 4.0–6.0) compared with 30 years and below age groups. Similarly, higher odds were found among the wealthiest wealth quintile than the poorest wealth quintile among men and women.

**Conclusion:**

The current study reported a high proportion of uncontrolled hypertension. Providing opportunistic health education during blood pressure monitoring, regular screening, and targeted interventions will not only help to reduce its prevalence but also reduce the risk of developing related health implications.

## Introduction

With rapid demographic transition and increasing proportions of the ageing population, Non-Communicable Diseases (NCDs) are expected to overwhelm the global health system. They are estimated to be responsible for the maximum out-of-pocket expenditure on health. Hypertension or high blood pressure (BP) is a preeminent cause of cardiovascular diseases (CVD), which are the leading causes of NCD-related morbidity and mortality. Just like the rest of the world, India has a high prevalence of hypertension [1]. Of the global 212 million Disability-Adjusted Life Years (DALYs) lost due to hypertension, 18% of the contribution was from India alone [2]. The most recent prevalence of hypertension in India is 28.1% [3]. What is even more concerning, according to the India Hypertension Control Initiative (IHCI) Progress Brief Report 2022, is that less than 10% of adults living with hypertension in India are known to have their BP under control [4]. Such an inability to achieve adequate BP control despite treatment is known as a state of uncontrolled hypertension [5]. Uncontrolled hypertension places the population at significant risk of CVD complications (including myocardial infarction, cerebrovascular accidents, cardiac arrhythmias, and renal failure) that are collectively responsible for over one-third of the total deaths in India [4]. Such a concerning situation necessitates urgent measures for controlling hypertension at the population level.

The development and progression of hypertension and emerging complications generally stem from multiple non-modifiable (e.g., sex, age, and genetic characteristics) and modifiable risk factors like tobacco, alcohol, inappropriate diet, lack of physical activity, increased sedentary behaviour, high Body mass index (BMI), environmental pollution and stress [6]. Most of these modifiable risk factors originate from human behaviour, thus known as behavioural risk factors. An individual’s intentional participation in some form of behaviour (like engagement with such risk factors) that involves potential negative consequences or losses (social, monetary, interpersonal) is termed unhealthy behaviour [7]. Data suggests a high prevalence of unhealthy behaviour among Indian adults. As per the National NCD Monitoring Survey (NNMS), 2017–18, the prevalence of risk factors associated with NCDs amongst adults (18–69 years), such as current tobacco use, current alcohol use, inadequate intake of fruits and/or vegetable intake and insufficient physical activity are 32.8%, 15.9%, 98.4%, and 41.3% respectively in India [8]. Regulation of such unhealthy behaviour is pivotal in controlling hypertension [9]. While Lifestyle changes decrease the impact of behavioural risk factors (like significantly better BP control among people living with hypertension), and unhealthy behaviours are linked with worsened outcomes. A higher prevalence of uncontrolled hypertension leads to higher mortality due to acute coronary events and cerebral vascular accidents [10].

Taking cognisance of the rising burden of NCDs and associated unhealthy behaviour, Goal 3 of the United Nations Sustainable Development aims to reduce premature mortality from NCDs by one-third by 2030. Also, it stresses the prevention and control of behavioural risk factors like tobacco and alcohol use to attain these goals. Population-based screening (PBS) was launched in 2016 to assess the NCD risk factors [6]. The National Health Policy (2017) also targets a 25% reduction in premature mortality from CVD, cancer, diabetes, or chronic respiratory diseases by 2025. The National Programme for Prevention and Control of NCDs (NP-NCD) focuses on early diagnosis, treatment, and follow-up of common NCDs, including hypertension [11]. However, there is still a gap between risk factors and disease management, as they are not addressed as an entity. So far, the program’s focus has been on screening and treatment, and lifestyle modifications are less prioritised [12]. Previous studies highlight the Government’s advocacy efforts against unhealthy behavioural risk factors that have been operating for a decade, but a knowledge gap exists in the community about such risk factors [13, 14]. Appel et al. reported that primary behavioural interventions focusing only on increasing physical activity, limiting dietary sodium intake, reducing alcohol consumption, and weight loss could drastically impact BP readings [13]. However, such behaviours are dynamic and vary over the lifespan, across cohorts, across settings, and over time. Thus, they need to be studied comprehensively from time to time to guide the policy better. Within this context, the Indian National Family Health Survey (NFHS), the adopted version of the Demographic Health Survey, collects data regarding hypertension and certain unhealthy behaviours. Using this opportunity, the present study aims to estimate the prevalence of uncontrolled hypertension among adult males and females, their association with unhealthy behaviours, and the likelihood of having uncontrolled hypertension in the presence of these unhealthy behaviours.

## Methodology

### Study setting

Globally, India is the seventh largest country geographically and the second-most populous country, with over 140 crores of population. India has twenty-eight states and eight union territories (UT). The states and UTs are further divided into districts as the administrative units. Districts are divided into census enumeration blocks (CEB)/wards in urban areas and villages/taluk in rural areas.

### Study design and study population

We did a secondary analysis of the fifth round of the NFHS data conducted during 2019–21. The NFHS-5 is a cross-sectional survey involving a two-stage cluster random sampling method using the population proportionate to the size technique. The International Institute for Population Sciences (IIPS, 2019) is designated a nodal agency for completing the survey under the stewardship of the Ministry of Health and Family Welfare, Government of India (GOI). The survey included all women in the reproductive age group (15–49 years) and men between 15–54 years of age. The survey’s nationally representative sample provides information on socioeconomic characteristics, height and weight measurements, nutrition, lifestyle, fertility, family planning, health knowledge, blood pressure, and random blood glucose values, along with information on various other characteristics. The present secondary analysis of NFHS-5 data was conducted between August to November 2022.

#### Sample size and sampling technique

**Fig 1:**
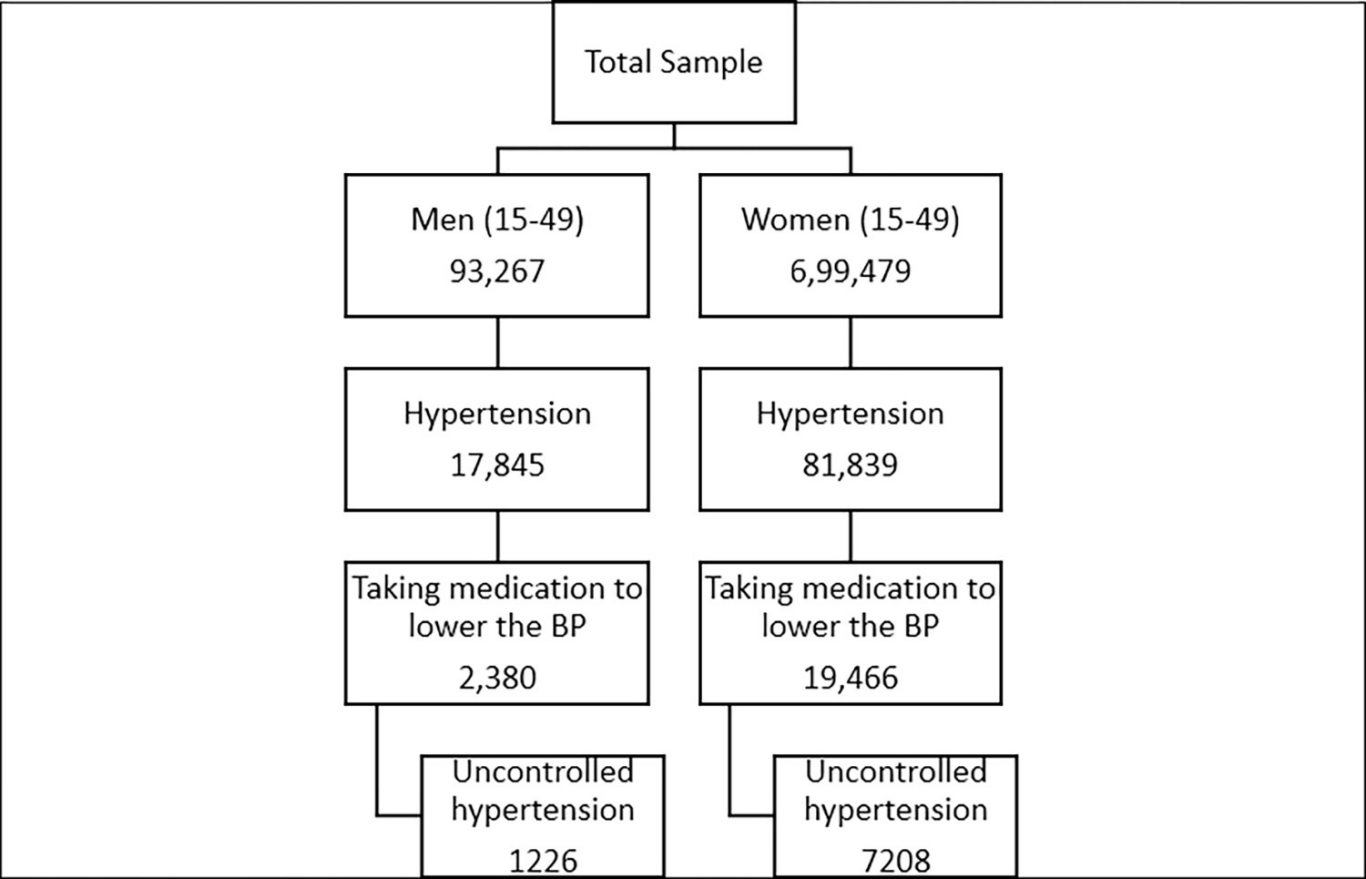
Flowchart depicting the sample selection process for the study analysis.

The NFHS-5 sample was self-weighted at the district level. It follows a two-stage sampling approach for selecting villages and Census Enumeration Blocks (CEBs) in rural areas and primary sampling units (PSUs) in urban areas. The 2011 Census data was used as the sampling frame. The survey followed the Probability Proportional to Size (PPS) sampling technique to choose villages/ CEBs within each rural/urban stratum. A detailed procedure of household selection is described in the NFHS -5 detailed report [15]. From these households, 748,176 women and 111,179 men of the reproductive age group were identified as eligible for the survey, of whom 724,115 women and 101,839 men completed the questionnaire with a response rate of 97% and 92% among women and men, respectively [15]. Of the total number of men and women in the reproductive age group, those with Diastolic Blood Pressure reading ≤20mm Hg and Systolic Blood Pressure reading ≥300mm Hg were excluded from the study, resulting in 93,267 men and 6,99,479 women. Among them, 2,380 men and 19,466 women were currently taking prescribed medication to lower blood pressure. This was taken as a base sample in the present study from whom people with uncontrolled hypertension were obtained using the operational definition (Fig 1). The data variables were extracted from the NFHS-5 India database, imported into the Microsoft Excel sheet for data cleaning, and analysed using STATA version 16.

#### Dependent variable

Uncontrolled Hypertension was the primary dependent variable. The study population diagnosed with hypertension and taking medications was first selected from the NFHS-5 variables (“SMB21/SB21: Currently taking prescribed medication to lower BP” for males and females, respectively). Among the population taking medicines for hypertension, the average of second and third systolic and diastolic readings was calculated. In the present study, people with average systolic blood pressure ≥ 140 mm Hg (or) average diastolic blood pressure ≥ 90 mm Hg were considered to have uncontrolled hypertension [16, 17].

#### Independent variables

Our primary independent variables were the presence or absence of unhealthy behaviours. For NCDs, the unhealthy behavioural risk factors stated by the World Health Organization include tobacco usage, alcohol intake, and physical inactivity [9]. In addition to these factors, indoor air pollution was included in the behavioural risk factors under NPCDCS [18]. In the present study, the choice of having health insurance coverage was included as one of the behavioural risk factors, considering its role in influencing the individual’s decision to seek treatment for their health condition and the burden posed by NCDs via out-of-pocket expenditure [19]. The presence of co-morbidities (diabetes mellitus and the presence of heart disease) were also considered unhealthy as they showcase the interplay of all unhealthy behaviours [20–22]. Given the well-documented link between the use of OC pills and hypertension, we included it as one of the behavioural risk factors for females [23]. The operational definitions for such variables were taken from the NFHS 5 as follows:

- Tobacco usage: Tobacco usage information among the population aged ≥15 years who currently use any form of tobacco, such as cigar, pipe, hookah, gutkha/paan masala with tobacco, khaini, paan with tobacco, other chewing tobacco, and snuff was gathered using variables mv463z/v463z for males and females respectively.
- Alcohol Usage: Alcohol usage patterns among the population aged >15 years were recorded using the variables sm619/s720 for males and females, respectively *(“Do you drink alcohol*?*” yes/no”*).
- High BMI: Physical activity was not directly assessed in NFHS, so BMI was considered a proxy indicator (underweight, normal, overweight, and obese) as per the standard WHO definition of overweight and obesity [24].
- Indoor air pollution was assessed using variables hv226/v161*(“What type of fuel does your household mainly use for cooking*?*”*) from the household questionnaire. All those households using LPG as the primary source for cooking were considered to be using clean fuel.
- Health insurance: The choice of having health insurance coverage was assessed based on their health insurance coverage using variable mv481/v481 (*“Are you covered by any health scheme or any health insurance*? *yes/no”*) for males and females, respectively.
- Co-morbidities: Information on co-morbidities (diabetes mellitus and presence of heart condition) for males and females was gathered from variables sm627a and sm627e/ s728a and s728e, respectively. Information regarding OC pills usage among females was obtained from the variable s321h.
- Unhealthy diet: A dietary diversity score ranging from 0 to 9 was calculated using variables such as milk, curd, pulses or beans, leafy vegetables, fruits, eggs, fish, chicken or meat, fried foods, and aerated drinks. A score of ‘0’ was considered to have no dietary diversity, and a score of ‘9’ was believed to have maximum diversity [25].

#### Covariates

These were selected based on an extensive literature review [16, 17, 21, 26–28]. The variables included for males and females were **age groups** (<30, 30-45and >45 years), **education** (no formal education, primary educated, secondary educated, and higher educated), religious beliefs (Hindu and non-Hindu), **household wealth index** (classified into five wealth quintiles), **current working status** (yes and no), **place of residence** (rural and urban). India was categorised into six geographical regions per the original analytical plan of NFHS-5. Region-wise data on uncontrolled hypertension was obtained by merging estimates from different states in the **six regions** as follows: North region(Jammu and Kashmir, Himachal Pradesh, Punjab, Chandigarh, Uttarakhand, Haryana, Delhi), Central region (Rajasthan, Uttar Pradesh, Chhattisgarh, and Madhya Pradesh); East region (West Bengal, Jharkhand, Odisha, and Bihar); North-East region (Sikkim, Arunachal Pradesh, Nagaland, Manipur, Mizoram, Tripura, Meghalaya, and Assam); West region (Gujarat, Maharashtra, Goa, Dadra & Nagar Haveli and Daman & Diu); and South region (Andhra Pradesh, Karnataka, Kerala, Tamil Nadu, and Puducherry).

### Data analysis

Our study utilised a sample of males and females from IAMR7CFL and IAIR7CFL datasets. Further, for men, the IAMR7FL file is merged with IAPR7CFL (IAPR7CFL is a ‘person file’ that consists of information on household members and their characteristics at the household level). However, for females, household-level information is available in the IAIR7CFL file. The study used descriptive analysis to determine the proportion of males and females with controlled and uncontrolled hypertension. We used descriptive statistics and bivariate statistics for analysis. Categorical variables were described in percentages and compared by Chi-square tests. Furthermore, multivariable logistic regression was applied to assess the factors associated with uncontrolled and controlled hypertension among males and females. The strength of association was examined by approximating adjusted odds ratios (OR) with their 95% CI. All statistical tests were two-tailed, and the cut-off of the significant level was defined as P < 0.05. In addition, all the estimates used national sample weight using the ‘svy’ command. Further, we have conducted a correlation matrix test followed by an interaction test for logistic regression. Due to insignificant results, we have included the tables in supporting information (S1–S4 Tables). All the analysis was done using STATA 16 (StataCorp, College Station, TX, USA). The map was created using Arc GIS version 10.2.

#### Ethical statement

This study is a secondary analysis of the NFHS-5 de-identified data set available publicly on the DHS Program website. It was ethically approved by the Institute’s Ethical Committee of the Postgraduate Institute of Medical Education and Research (PGIMER), Chandigarh, vast letter number IEC-08/2022-2535, dated 17.08.2022.

## Results

The proportion of males and females with uncontrolled hypertension who used prescribed medication was 49.5% (95% CI: 45.5–53.4) and 36.8% (95% CI: 35.8–37.8), respectively. Uncontrolled hypertension was higher among older age groups in males (56%) and females (51%). Among both males and females, uncontrolled hypertension was highest among the widowed, divorced/ separated (76%, 48%, respectively), followed by the currently married group (52%, 38%, respectively). There was no variation in the proportion of uncontrolled hypertension across educational levels among males. Among females, the proportion of uncontrolled hypertension decreased with increasing educational levels. The proportion of uncontrolled hypertension among currently working males was 52% (95% CI: 48–56.5), and now, working females were 37% (95% CI: 31.2–42.6). There was a significant difference in the proportion of uncontrolled hypertension among urban males (51%) and rural males (48%). Similarly, there was a substantial difference in the proportion of uncontrolled hypertension among urban females (41%) and rural females (34%). The prevalence of uncontrolled hypertension was noted with an increase in the wealth index from poorest to richest among males and females **(Table 1)**.

**Table 1:** Distribution of sociodemographic variables among the population with uncontrolled and controlled hypertension by males and females participating in the fifth round of the National Family Health Survey India (2019–21).

	Hypertension in males				Hypertension in females			
	Total sample	Uncontrolled hypertension			Total sample	Uncontrolled hypertension		
		Un-weighted counts	Weighted % (95%CI)	P-Value		Un-weighted counts	Weighted % (95%CI)	P-Value
**Total**	2380	1,226	49·5 (45·5–53·4)		19,466	7,208	36·8(35.8–37.8)	
**Age groups**								
<30	337	52	17.0(10·7–25·2)	<0**·**001	4458	463	10.0(8·6–11)	<0**·**001
30–45	989	520	52.0(45.8–58.4)		10400	4339	41.0(40.0–42.7)	
>45	1054	654	56.0(50·5–61·7)		4608	2406	51.0(48·6–52·7)	
**marital status**								
Currently unmarried	297	68	22.0(15·4–30·8)	<0**·**001	1845	202	11.0(8·8–13)	<0**·**001
Currently Married	2032	1130	52.0(48·1–56·7)		16081	6267	38.0(37·2–39·4)	
Widowed/divorced/separated	51	28	76.0(55·2–88·6)		1540	739	48.0(44·4–51·2)	
**Educational level**								
No education	243	139	57.0(45·7–66·9)	0**·**131	5540	2310	42.0(40–43·6)	<0**·**001
Primary	312	168	48.0(38·9–57·2)		2841	1155	40.0(37·3–42·3)	
Secondary	1346	669	51.0(45·8–56)		9088	3187	35.0(33·8–36·6)	
Higher	477	248	43.0(34·3–52·2)		1997	556	27.0(24·1–29·8)	
**Currently working**								
No	364	139	32.0(24·5–41)	<0**·**001	2115	768	38.0(34·7–40·4)	0**·**260
Yes	2016	1087	52.0(48–56·5)		780	301	37.0(31·2–42·6)	
**Religion**								
Hindu	1742	892	49.0(44·4–53·2)	0**·**62	13622	4873	36.0(34·5–36·7)	<0**·**001
Non-Hindu	638	334	52.0(43·7–60·5)		5844	2335	41.0(38·7–43)	
**Ethnicity**								
NO caste/tribe	134	64	47.0(32·1–61·8)	0**·**383	11465	4242	37.0(35·7–38)	0**·**385
Caste/tribe	2229	1151	50.0(45·5–53·6)		6539	2377	35.0(33·3–36·7)	
**Type of place of residence**								
Urban	902	504	51.0(44·7–58·2)	<0**·**001	5930	2417	41.0(39·6–43·1)	<0**·**001
Rural	1478	722	48.0(43·3–52·3)		13536	4791	34.0(33–35·2)	
**Wealth index**								
Poorest	256	97	30.0(21·1–41·4)	<0**·**001	3014	817	26.0(23·5–28·0)	<0**·**001
Poorer	417	192	43.0(34·8–52·3)		3654	1245	32.0(29·9–34·1)	
Middle	474	229	49.0(39·8–57·6)		4139	1601	38.0(36·2–40·5)	
Richer	594	351	59.0(52·5–65·2)		4342	1777	40.0(38·4–42·4)	
Richest	639	357	50.0(41·7–57·7)		4317	1768	42.0(39·8–44)	

Note: The number of missing cases varied across background characteristics.

Behavioural characteristics like alcohol consumption, clean fuel usage, and high BMI were significantly associated with uncontrolled hypertension among males. Tobacco usage, alcohol consumption, coverage by health insurance, presence of diabetes, heart disease, usage of clean fuel, and high BMI were the behavioural characteristics significantly associated with uncontrolled hypertension among females **(Table 2)**.

**Table 2:** Weighted Percentage of males and females with controlled and uncontrolled hypertension by some selected behavioural characteristics who participated in the fifth round of the National Family Health Survey India (2019–21).

	Hypertension in males				Hypertension in females			
	Total sample	Uncontrolled hypertension			Total sample	Uncontrolled hypertension		
		Un-weighted counts	Weighted % (95%CI)	P-Value		Un-weighted counts	Weighted % (95%CI)	P-Value
**Total**	2380	1,226	49·5( 45·5–53·4)		19,466	7,208	36·8(35.8–37.8)	
**High BMI**								
Yes	290	182	53·9(41·1–66·1)	<0·001	3,275	1,663	49·3(46·8–51·7)	<0·001
No	1,466	659	44·6(39·7–49·6)		12,045	3,532	28·2(27·1–29·4)	
**Diabetes**								
No	2,088	1,062	50·8(46·6–55)	0·214	17,467	6,174	34·9(33·9–35·9)	<0·001
Yes	262	146	40·1(30·2–50·9)	0·761	1,756	921	51·9(48·6–55·2)	
**Heart Disease**								
No	2,292	1,184	50·5(46·6–54·3)		18,830	6,943	36·7(35·7–37·6)	0·036
Yes	78	37	33·3(16·9–55·1)		529	217	39·8(34·1–45·7)	
**Tobacco use in any form**				0·843				
No	1,348	692	48·4(43·4–53·3)		17,878	6,534	36·6(35·7–37·6)	<0·001
Yes	1,032	534	51(44·9–57)		1,588	674	39·8(36·1–43·7)	
**Alcohol**								
No	1,634	786	47(42·5–51·5)	<0·001	19,051	7,007	36·7(35·8–37·7)	<0·001
Yes	746	440	55·1(48·3–61·6)		415	201	46·4(39·3–53·6)	
**Dietary diversity**								
Absent	23	14	65·1(29·8–89·1)	0·367	186	57	29·2(21·3–38·6)	0·07
Present	2,357	1,212	49·4(45·5–53·4)		19,280	7,151	36·9(35·9–37·8)	
**Covered by health insurance**								
No	1,364	683	46·9(41·7–52·1)	0·104	12,703	4,586	35·8(34·6–37)	<0.001
Yes	1,016	543	52·9(47·1–58·6)		6,763	2,622	38·7(37·2–40·3)	
**Cooking Fuel**								
Polluting	748	331	41·4(35·2–47·9)	<0·001	7,641	2,416	30·3(28·9–31·8)	<0·001
Clean	1,632	895	52·3(47·6–56·9)		11,825	4,792	40·4(39·1–41·7)	
**Oral contraceptive pills use**								
No	··	··	·	··	17,605	6,982	39.0(37·9–40)	0·085
Yes	··	··	·		133	43	43.0(30·7–56·1)	

Note: The number of missing cases varies across behavioural characteristics

**Fig 2:**
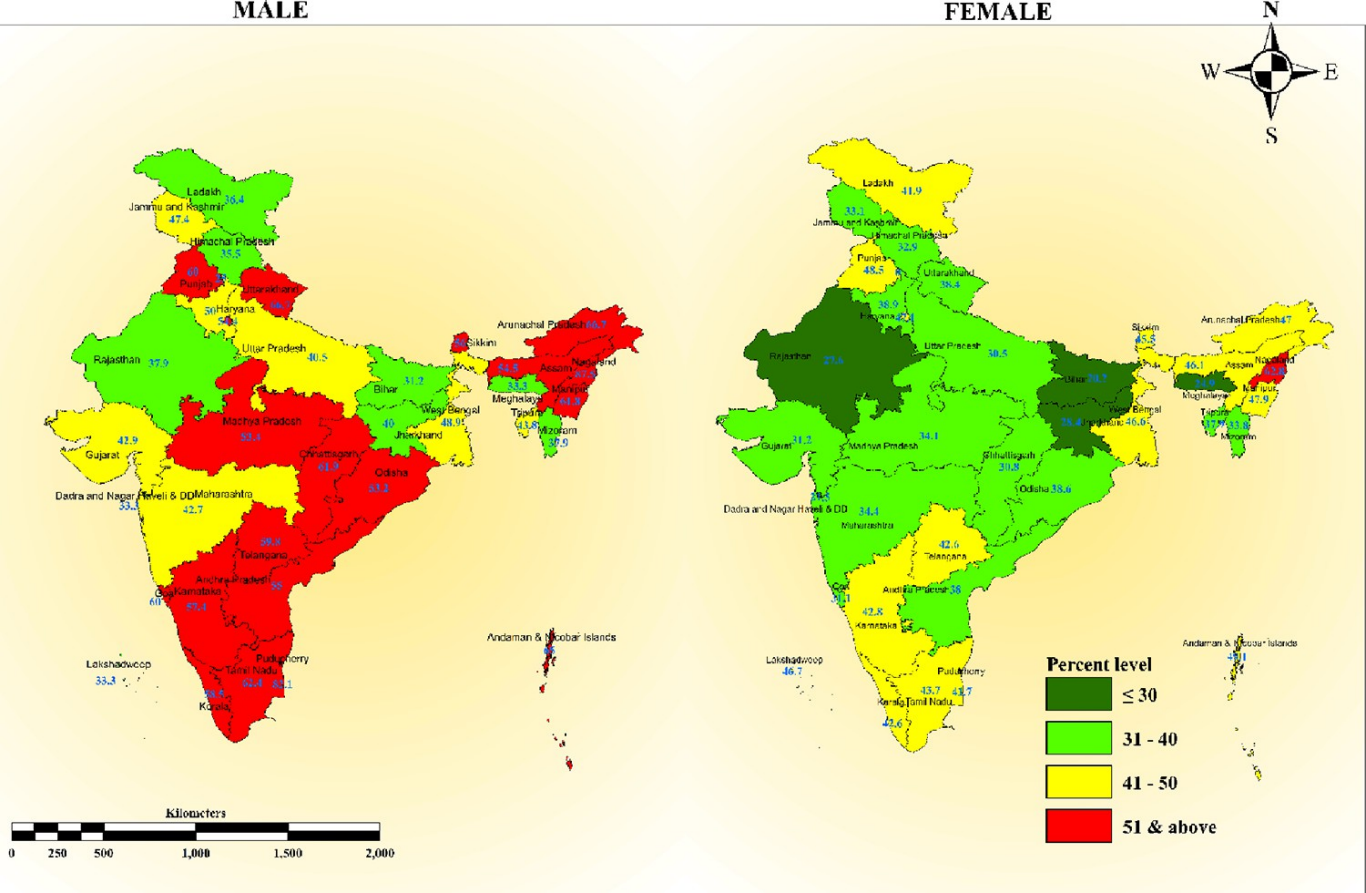
Map showing a state-wise prevalence of uncontrolled hypertension among males and females who participated in the fifth round of the National Family Health Survey India (2019–21).

Among males, >50% of uncontrolled hypertension was observed in Punjab and Uttarakhand from the Northern region, Arunachal Pradesh, Assam, Nagaland, and Manipur from the North-eastern region, Orissa and Sikkim from the Eastern region, Madhya Pradesh and Chhattisgarh from the central region. Among females, >50% prevalence of uncontrolled hypertension was observed in Nagaland from the North-Eastern part of India **(Fig 2)**.

On bivariate analysis, Age, marital status, education (among females), currently working population (among males), urban residents, population belonging to richer wealth quintiles, with high BMI, diabetics (among females), tobacco users (among females), alcohol users, health insurance coverage (among females) and clean cooking fuel usage had significant association with uncontrolled hypertension. On adjusting for other variables, age, marital status (among males), currently working population (among males), urban residents (among females), tobacco users (among females), alcohol users (among males) and high BMI obtained significant association with uncontrolled hypertension **(Table 3)**.

**Table 3:** Predictors of uncontrolled blood pressure among hypertensive males and females, who participated in the fifth round of the National Family Health Survey India (2019–21).

	Males		Females	
	Uncontrolled hypertension		Uncontrolled hypertension	
	Adjusted Odds ratio (95% CI)	P-value (Global)	Adjusted Odds ratio(95% CI)	P-value (Global)
**Age (Completed years)**				
<30	**Ref**	0**·**000	**Ref**	<0.001
30–45	4·90(3·1–7·9)		4·04(2·7–5·9)	
>45	7·60(4·6–12·3)		6·08(4**·**0–9**·**0)	
**Marital status**				
Single/ Currently Not Married	**Ref**	0**·**025	**Ref**	0**·**430
Currently Married	1·05(0·6–1·6)		2·76(0·34–22·2)	
Widowed/divorced/separated	1·08(0·5–2·4)		3·01(0·4–24)	
**Educational level**				
No education	**Ref**	0**·**877	**Ref**	0**·**802
Primary	0·91(0·6–1·4)		1·08(0·8–1·5)	
Secondary	0·87(0·6–1·2)		0·95(0·8–1·2)	
Higher	1·08(0·7–1·6)		1·21(0·8–1·8)	
**Currently working**				
No	**Ref**	0**·**007	**Ref**	0**·**704
Yes	1·35(0·99–1·8)		0·98(0·78–1·22)	
**Religion**				
Hindu	**Ref**	0**·**153	**Ref**	0**·**013
Non-Hindu	1·18(0·9–1·5)		1·33(1–1·7)	
**Social Caste**				
NO caste/tribe	**Ref**	0**·**595	**Ref**	0**·**616
Caste/tribe	0·86(0·5–1·3)		0·95(0·7–1·1)	
**Place of residence**				
Urban	**Ref**	0**·**745	**Ref**	0**·**004
Rural	0·95(0·7–1·2)		1·44(1·1–1·8)	
**Wealth index**				
Poorest	**Ref**	0**·**139	**Ref**	0**·**474
Poorer	1·34(0·9–2)		1·47(1–2)	
Middle	1·39(0·9–2)		1·31(0·9–1·9)	
Richer	1·74(1·1–2·7)		1·32(0·8–2)	
Richest	1·42(0·87–2·3)		1·28(0·8–2)	
**Tobacco use in any form**				
No	**Ref**	0**·**796	**Ref**	0**·**040
Yes	0·99(0·8–1·2)		0·70(0·5–0·9)	
**Alcohol use**				
No	**Ref**	0**·**016	**Ref**	0**·**343
Yes	1·34(1·07–1·69)		1·34(0·71–2·52)	
**Dietary diversity**				
Absent	**Ref**	0**·**046	**Ref**	0**·**415
Present	0·35(0·13–0·97)		1·89(0·5–7·2)	
**Covered by health insurance**				
No	**Ref**	0**·**789	**Ref**	0**·**736
Yes	1·02(0·8–1·26)		1·02(0·8–1·3)	
**Diabetes**				
No	**Ref**	0**·**935	**Ref**	0**·**350
Yes	0·95(0·7–1·3)		1·35(0·97–1·88)	
**Heart Disease**				
No	**Ref**	0**·**803	**Ref**	0**·**745
Yes	0·82(0·5–1·45)		1·34(0·7–2·5)	
**Cooking Fuel**				
Clean	**Ref**	0**·**390	**Ref**	0**·**122
Polluting	0·94(0·70–1·26)		0·84(0·65–1·10)	
**Oral contraceptive pills use**				
No	-		**Ref**	0**·**751
Yes	-		0·72(0·14–3·8)	
**High BMI**				
Non- Obese	**Ref**	0**·**005	**Ref**	0**·**000
Obese	1·48(1·11–1·95)		1·90(1·49–2·43)	

## Discussion

The present study estimated the proportion of uncontrolled hypertension among adults taking prescribed medication and having behavioural risk factors. Significant findings emerge from our study that call for concerted efforts to realise our hypertension-related goals. First, about half of the participants had uncontrolled hypertension with significant association across different socio-demographic strata. Second, certain unhealthy behaviours were significantly associated with uncontrolled hypertension with little gender-based differences. Lastly, certain unhealthy behaviours depicted a significantly increased likelihood of having uncontrolled hypertension.

The high prevalence of uncontrolled hypertension in India, as observed in our study, is a cause of concern. Nearly similar proportions of uncontrolled hypertension were observed in studies from different countries using the exact operational definition for uncontrolled hypertension [10, 16]. In comparison, a study from Zimbabwe reported a higher prevalence (67.2%) than the present study of uncontrolled hypertension and used the same operational definitions [17]. The geographical disparities in the prevalence of uncontrolled hypertension prevalence can be attributed to baseline education and socio-economic status that are known to affect the health literacy of the population and health-seeking behaviour subsequently [29, 30]. Other factors leading to uncontrolled status may include lack of medication adherence, non-availability of prescribed medication, cost of medicines or even treatment inertia of the health practitioner [31]. Hospital-based studies report a lower prevalence of uncontrolled hypertension, but such studies are subjected to selection bias and lack generalisability [26].

We observed significant variations in the prevalence of uncontrolled as per socio-demographic characteristics. Higher age groups, widowed/divorced/separated individuals, people with more than secondary level education, currently working population, people residing in urban areas, and those belonging to richer wealth quintiles were observed to have significantly higher proportions of uncontrolled hypertension in the present study. Age is a non-modifiable risk factor for all NCDs, and genetics plays a significant role. The asymptomatic nature of the disease in most cases and lifelong medication increase mental fatigue, leading to loss of medication adherence and making hypertension uncontrolled. The present study supports it by depicting the significant increase in the proportion of uncontrolled hypertension with increasing age among both males and females. As a response, steps towards early diagnosis of NCDs and thus preventing associated complications are already in place by population-based screening of all individuals aged above 30 years. Similar to this study, the widowed/divorced/separated population had a higher proportion of uncontrolled hypertension when compared to married people in national-level surveys done in Zimbabwe and Saudi Arabia [17, 27]. In contrast to the present study, the married population had a higher proportion of uncontrolled hypertension in a few national and monocentric studies [16, 21, 28]. Literature on marital status and hypertension is inconclusive. While some studies attribute uncontrolled hypertension in some couples to intentional engagement in risk factors together, others report inculcating healthy behaviour through emotional support and motivation [19, 32]. Unlike the present study, previous studies report higher uncontrolled hypertension in illiterate people with fewer years of education from poor socio-economic backgrounds [17, 21, 27, 28, 31]. Many times, more educated and affluent patients tend to take their disease casually due to other pressing priorities. Also, emerging reports suggest a decreasing rich-poor gap in NCDs and their risk factors [33]. These decreasing disparities necessitate a more in-depth review of the health advocacy policies that have concentrated on the poor and the vulnerable section of the society. Further, the working population was observed to have a higher proportion of uncontrolled hypertension in a study done in rural India and a national-level study done in Thailand, similar to the present study [26, 28]. Though contrasting findings have been reported from other studies, work-related stress, less time for physical activities and more exposure to smoking and alcohol have been related to work-related hypertension [17, 21]. On the other hand, poor socio-economic status makes people dependent on the health system for their treatment adherence among the non-working population. The possible reason behind this variation is the usage of different socio-economic scales in different studies.

In the present study, a higher proportion of uncontrolled hypertension was observed among people with **diabetes** in both males and females. Though the association was not statistically significant, this finding was consonant with the findings in a few previous studies [17, 22, 27]. Diabetes is frequently co-existing with hypertension due to common risk factors [34, 35]. Further, insulin has been shown to stimulate the sympathetic nervous system, increase renal sodium retention, modulate cation transport, and induce hypertrophy of vascular smooth muscle [36]. Such a scenario makes it pertinent to screen for hypertension while managing diabetes and vice versa. In this study, hypertensives without **heart disease** were at higher risk of uncontrolled hypertension among males. This finding was also in line with the findings of a few other studies [21, 22]. People without co-morbid complaints usually have less health-seeking behaviour and poor motivation for treatment adherence coupled with lower awareness regarding complications of uncontrolled hypertension, which might be the probable reason for their uncontrolled hypertension status. Among females, uncontrolled hypertension was slightly higher among those with heart disease when compared to those without heart disease which is in line with existing evidence. Maintaining blood pressure under control among patients with co-morbidities is challenging [20].

**Obese people** had a significantly higher proportion of uncontrolled hypertension among both males and females in the present study. This observation has also been demonstrated in various other studies [10, 16, 17, 21, 26]. Literature also establishes that obese patients on anti-hypertensive medication are less likely to reach recommended targets when compared to their counterparts with normal weight. Modest weight loss will reverse blood pressure elevations and favour obesity-related cardiovascular risk factors such as diabetes and lipidaemias [17]. Health education regarding the benefits of weight loss to all hypertensive populations might result in a lower proportion of uncontrolled hypertension among the already diagnosed hypertensive population. **Non-users** of tobacco among females and alcohol users among males had significantly higher uncontrolled hypertension. Tobacco use is an established risk factor for NCDs, and thus, further in-depth studies looking into dose-response relationships and causality are necessary as supportive evidence for uncontrolled hypertension among non-users of tobacco. An association between **alcohol** consumption and uncontrolled hypertension has also been demonstrated in various national-level studies in India and other countries, supporting the recommendation for limiting alcohol consumption [10, 16, 28]. In our study, the population using clean fuel as a primary source of cooking and those with health insurance coverage had higher uncontrolled hypertension. Though no significant association was observed for these findings, there is evidence symbolising clean fuel usage among the population with a better wealth index. Further, hypertension disproportionately affects the wealthy segment of our country. Hence, clean fuel usage can be considered a proxy indicator for the wealth index associated with uncontrolled hypertension [37].

There is mixed evidence about the association between **health insurance coverage** and risky behaviours. Consistent with our findings, Dave et al., in their study, concluded that obtaining health insurance reduces prevention and increases unhealthy behaviours [32]. This increase in unhealthy behaviours plays a direct role in uncontrolled hypertension, as observed in the present study. Further studies will be needed to explore the effect of adherence to treatment and understanding of the disease condition on BP control to confirm our findings. Uncontrolled hypertension and its associated complications also have enormous economic costs for patients and their families, health systems and national economies. The cost of acute hospital and outpatient care for heart attacks and strokes caused by uncontrolled hypertension is overwhelming for the health systems as well. People living with such conditions incur direct medical expenses and lost wages, often in their prime working years, which can be impoverishing for entire families.

There are a few strengths and limitations of the study that should be acknowledged. While our estimates ensure generalizability as the data has been generated from a national survey that is collected following a robust methodology, a few limitations of this study should be acknowledged. The current secondary data analysis is based upon the data retrieved from the cross-sectional NFHS survey, where individual BP and related measures were available for the age group of 15–49 years only. Hence, measuring the current treatment uptake in the higher age groups who are also most commonly affected by hypertension could not be done. Therefore, the burden of uncontrolled hypertension is not a true representation for adults in India. The analysis is limited to the finite number of variables included in the survey, while many other factors may affect the outcomes of the research question, like the availability of medication, treatment inertia of the treating doctors, and awareness about the disease and its complications. It is highly recommended that future studies include more of these variables. The study’s cross-sectional nature limits us from commenting on the temporal association between the risk factors and the diseases. A further limitation of this study is that we did not include women per their pregnancy or lactation status when blood pressure fluctuates during this time.

Policy implications for hypertension control extend beyond managing the condition itself, as hypertension often exacerbates other health issues such as heart disease, stroke, and kidney failure, leading to a significant economic burden. Effective policies should emphasize comprehensive prevention strategies, including public education on lifestyle modifications and regular blood pressure screenings. Integrating hypertension management into broader healthcare policies can improve overall health outcomes and reduce the incidence of comorbid conditions. Additionally, ensuring equitable access to affordable medications and healthcare services can alleviate long-term healthcare costs. By addressing the multifaceted impacts of hypertension, such policies can mitigate its economic burden on both individuals and healthcare systems.

To conclude, despite taking prescribed medication for hypertension, nearly half of our men and one-third of women depicted uncontrolled hypertension. Hence, Tobacco usage, alcohol consumption, obesity, indoor air pollution, and the presence of co-morbid conditions should be given utmost priority during patient management. Emphasis should be given regarding hazards of non-adherence to hypertensive medication and the importance of lifestyle modifications while managing a population any of these factors should be observed to be significant unhealthy behaviours. This helps us achieve one of the nine voluntary global targets for preventing and controlling NCDs by 2025 against a 2010 baseline, i.e. “25% relative reduction in the prevalence of raised blood pressure”. Increasing the percentage of people whose hypertension is under control globally to 50% would prevent 76 million deaths between 2023 and 2050. Other characteristics like higher age groups, widowed/divorced/separated individuals, people with more than secondary level education (among females), currently working population (among males), people residing in urban areas, diabetics, and those belonging to wealthier wealth quintiles were identified as strong predictors of uncontrolled hypertension. Providing opportunistic health education during blood pressure monitoring, regular screening, and targeted interventions focusing on middle-aged hypertensives capable of accepting lifestyle changes will help reduce its prevalence and the risk of developing related health implications. Also, cohort studies can further assess the temporal association of the identified factors using this as baseline data.

## Supporting information

S1 Table Correlation matrix between all the predictor variables selected in the study for males. Only highlighted values selected for the interaction test due to strong correlation between them.
(DOCX)

S2 Table Adjusted effect of uncontrolled hypertension among males with interaction results. *Estimated values are adjusted with Education, working status, religion, ethnicity, tobacco use, alcohol, dietary diversity, covered with health insurance, diabetic, heart disease, and body mass index.
(DOCX)

S3 Table Correlation matrix between all the predictor variables selected in the study for females. Only highlighted values selected for the interaction test due to strong correlation between them.
(DOCX)

S4 Table Adjusted effect of uncontrolled hypertension among females with interaction results. *Estimated values are adjusted with age groups, marital status, current working status, religion, ethnicity, tobacco use, alcohol use, dietary diversity, covered health insurance, diabetic, heart disease, OCP use, and BMI.
(DOCX)
