# Supplementary material for: Unhealthy behaviours associated with uncontrolled hypertension among adults in India- Insights from a national survey
Source: PLoS One. 2025 Jan 17;20(1):e0310099. doi: 10.1371/journal.pone.0310099 (PMC11741589; doi:10.1371/journal.pone.0310099)
Supplement: S1 Table — Only highlighted values selected for the interaction test due to strong correlation between them. (DOCX) [file pone.0310099.s001.docx]

| **S1 Table a1: Correlation matrix between all the predictor variables selected in the study for males** | | | | | | | | | | | | | | | | |
| --- | --- | --- | --- | --- | --- | --- | --- | --- | --- | --- | --- | --- | --- | --- | --- | --- |
|  | **Age groups** | **marital status** | **Educational level** | **Currently working** | **Religion** | **Ethnicity** | **Type of place of residence** | **Wealth index** | **Tobacco use in any form** | **Alcohol use** | **Dietary diversity** | **Covered by health insurance** | **Diabetic** | **Heart Disease** | **Cooking Fuel** | **BMI** |
|  |  |  |  |  |  |  |  |  |  |  |  |  |  |  |  |  |
| **Age groups** | 1 |  |  |  |  |  |  |  |  |  |  |  |  |  |  |  |
| **marital status** | 0**·**559 | 1 |  |  |  |  |  |  |  |  |  |  |  |  |  |  |
| **Educational level** | -0**·**1263 | -0**·**1152 | 1 |  |  |  |  |  |  |  |  |  |  |  |  |  |
| **Currently working** | 0**·**2221 | 0**·**3088 | -0**·**0284 | 1 |  |  |  |  |  |  |  |  |  |  |  |  |
| **Religion** | -0**·**0875 | -0**·**0608 | -0**·**097 | -0**·**042 | 1 |  |  |  |  |  |  |  |  |  |  |  |
| **Ethnicity** | 0**·**0492 | 0**·**0459 | 0**·**0622 | -0**·**005 | -0**·**1763 | 1 |  |  |  |  |  |  |  |  |  |  |
| **Type of place of residence** | -0**·**0894 | -0**·**0343 | -0**·**1683 | -0**·**0242 | 0**·**0537 | -0**·**0449 | 1 |  |  |  |  |  |  |  |  |  |
| **Wealth index** | 0**·**1556 | 0**·**0674 | 0**·**3659 | 0**·**0703 | -0**·**1123 | 0**·**1037 | -0**·**4879 | 1 |  |  |  |  |  |  |  |  |
| **Tobacco use in any form** | -0**·**0899 | -0**·**1433 | 0**·**1865 | -0**·**069 | 0**·**0077 | 0**·**0748 | -0**·**0651 | 0**·**198 | 1 |  |  |  |  |  |  |  |
| **Alcohol use** | 0**·**1199 | 0**·**1239 | -0**·**0508 | 0**·**0964 | -0**·**1165 | 0**·**0578 | -0**·**0391 | 0**·**0423 | -0**·**2528 | 1 |  |  |  |  |  |  |
| **Dietary diversity** | 0**·**0419 | -0 **·**0064 | 0**·**0587 | -0**·**0159 | -0**·**0615 | 0**·**0435 | -0**·**0336 | 0**·**0835 | 0**·**0609 | -0**·**0018 | 1 |  |  |  |  |  |
| **Covered by health insurance** | 0**·**0889 | 0**·**1078 | -0**·**003 | 0**·**0647 | -0**·**0965 | -0**·**0215 | 0**·**0211 | 0**·**0071 | 0**·**0242 | 0**·**0394 | 0**·**0128 | 1 |  |  |  |  |
| **Diabetic** | 0**·**1027 | 0**·**0666 | -0**·**0541 | 0**·**008 | 0**·**0361 | -0**·**0198 | -0**·**0289 | -0**·**0005 | -0**·**0432 | 0**·**0477 | -0**·**0293 | 0**·**0152 | 1 |  |  |  |
| **Heart Disease** | 0**·**0076 | 0**·**009 | -0**·**0316 | -0**·**0086 | 0**·**0079 | 0**·**005 | 0**·**0236 | -0**·**0504 | -0**·**0225 | -0**·**0052 | 0**·**0122 | 0**·**0342 | 0**·**0832 | 1 |  |  |
| **Cooking Fuel** | 0**·**111 | 0**·**062 | 0**·**2453 | 0**·**0804 | -0**·**079 | 0**·**0626 | -0**·**41 | 0**·**6588 | 0**·**1297 | 0**·**1136 | 0**·**052 | 0**·**012 | 0**·**0249 | -0**·**0348 | 1 |  |
| **BMI** | -0**·**1459 | -0**·**1374 | -0**·**1043 | -0**·**1025 | 0**·**0278 | -0**·**0533 | 0**·**1347 | -0 **·**2485 | -0**·**0967 | -0**·**0278 | -0**·**0318 | -0**·**0358 | -0**·**0133 | -0**·**0041 | -0**·**1656 | 1 |
| Note- only highlighted values selected for the interaction test due to strong correlation between them | | | | | | | | | | | | | | | | |
