# Supplementary material for: Unhealthy behaviours associated with uncontrolled hypertension among adults in India- Insights from a national survey
Source: PLoS One. 2025 Jan 17;20(1):e0310099. doi: 10.1371/journal.pone.0310099 (PMC11741589; doi:10.1371/journal.pone.0310099)
Supplement: S2 Table — *Estimated values are adjusted with Education, working status, religion, ethnicity, tobacco use, alcohol, dietary diversity, covered with health insurance, diabetic, heart disease, and body mass index. (DOCX) [file pone.0310099.s002.docx]

| **S2 Table a2 : Adjusted effect of uncontrolled hypertension among males with interaction results** | | | |
| --- | --- | --- | --- |
| **Age vs Marital Status** | Odds ratio | P-value | 95% CI |
| <30 vs ever married (ref) |  |  |  |
| 30-45 vs Never married | 0**·**79 | 0**·**639 | 0**·**28-2**·**15 |
| >45 vs Never married | 0**·**85 | 0**·**831 | 0**·**18-3**·**90 |
| **Cooking fuel vs Wealth Index** |  |  |  |
| Polluting vs poorest (ref) |  |  |  |
| Clean vs poorer | 1**·**05 | 0**·**944 | 0**·**28-3**·**87 |
| Clean vs middle | 0**·**90 | 0**·**868 | 0**·**24-3 **·**28 |
| Clean vs richer | 1**·**10 | 0**·**891 | 0**·**28-4**·**32 |
| Clean vs Richest | 0**·**94 | 0**·**949 | 0**·**14-6**·**17 |
| **Walth Index vs Residence** |  |  |  |
| Poorest vs urban (ref) |  |  |  |
| Poorer vs rural | 1**·**28 | 0**·**818 | 0**·**15-10**·**64 |
| Middle vs rural | 1**·**36 | 0**·**772 | 0**·**17-11**·**15 |
| Richer vs rural | 1**·**29 | 0**·**816 | 0**·**15-10**·**61 |
| Richest vs rural | 1**·**40 | 0 **·**757 | 0**·**16-12 **·**04 |
| **Cooking fuel vs Residence** |  |  |  |
| Polluting vs Urban (Ref) |  |  |  |
| Clean vs Rural | 1 **·**04 | 0**·**923 | 0 **·**47-2 **·**31 |
| *Estimated values are adjusted with Education, working status, religion, ethnicity, tobacco use, alcohol, dietary diversity, covered with health insurance, diabetic, heart disease, and body mass index | | | |
