# Supplementary material for: Unhealthy behaviours associated with uncontrolled hypertension among adults in India- Insights from a national survey
Source: PLoS One. 2025 Jan 17;20(1):e0310099. doi: 10.1371/journal.pone.0310099 (PMC11741589; doi:10.1371/journal.pone.0310099)
Supplement: S3 Table — Only highlighted values selected for the interaction test due to strong correlation between them. (DOCX) [file pone.0310099.s003.docx]

| **S3 Table b1: Correlation matrix between all the predictor variables selected in the study for females** | | | | | | | | | | | | | | | | | |
| --- | --- | --- | --- | --- | --- | --- | --- | --- | --- | --- | --- | --- | --- | --- | --- | --- | --- |
|  | Age groups | marital status | Educational level | Currently working | Religion | Ethnicity | Type of place of residence | Wealth index | Tobacco use in any form | Alcohol use | Dietary diversity | Covered by health insurance | Diabetic | Heart Disease | Cooking Fuel | OCP use | BMI |
|  |  |  |  |  |  |  |  |  |  |  |  |  |  |  |  |  |  |
| Age groups | 1·0000 |  |  |  |  |  |  |  |  |  |  |  |  |  |  |  |  |
| marital status | 0·1988 | 1·0000 |  |  |  |  |  |  |  |  |  |  |  |  |  |  |  |
| Educational level | -0·1609 | -0·1170 | 1·0000 |  |  |  |  |  |  |  |  |  |  |  |  |  |  |
| Currently working | 0·0767 | 0·0664 | -0·0676 | 1·0000 |  |  |  |  |  |  |  |  |  |  |  |  |  |
| Religion | 0·0067 | -0·0189 | -0·0426 | -0·0305 | 1·0000 |  |  |  |  |  |  |  |  |  |  |  |  |
| Ethnicity | -0·0907 | -0·0004 | -0·1206 | 0·0938 | 0·0807 | 1·0000 |  |  |  |  |  |  |  |  |  |  |  |
| Type of place of residence | -0·1233 | -0·0292 | -0·2386 | 0·0394 | -0 ·0237 | 0·1057 | 1·0000 |  |  |  |  |  |  |  |  |  |  |
| Wealth index | 0·1970 | 0·0189 | 0·3930 | -0·0587 | 0·0067 | -0·2882 | -0·4849 | 1·0000 |  |  |  |  |  |  |  |  |  |
| Tobacco use in any form | -0·0714 | -0·0325 | 0·1132 | -0·0655 | -0·1199 | -0·1544 | -0·0299 | 0·1814 | 1·0000 |  |  |  |  |  |  |  |  |
| Alcohol use | 0·0180 | 0·0091 | -0·0632 | 0·0800 | 0·0511 | 0·0975 | 0·0594 | -0·0971 | -0 ·1360 | 1·0000 |  |  |  |  |  |  |  |
| Dietary diversity | 0·0194 | 0·0236 | 0·0271 | 0·0111 | -0·0360 | -0 ·0281 | 0·0286 | 0·0103 | 0·0167 | 0·0131 | 1 ·0000 |  |  |  |  |  |  |
| Covered by health insurance | 0·1134 | 0·0137 | -0·0485 | 0·0977 | -0·0690 | 0·0166 | -0·0145 | 0·0331 | -0 ·0473 | 0·0146 | -0·0160 | 1 ·0000 |  |  |  |  |  |
| Diabetic | 0·0154 | 0·0090 | 0·0177 | 0·0470 | 0 ·1161 | 0·0623 | -0·0262 | -0·0129 | -0·0567 | 0·0005 | 0·0103 | -0·0018 | 1.0000 |  |  |  |  |
| Heart Disease | 0·0170 | 0·0165 | 0·0212 | 0·0180 | 0·0146 | 0·0415 | -0·0051 | 0·0050 | -0·0334 | 0·0238 | 0 ·0011 | 0 ·0039 | 0.2893 | 1.0000 |  |  |  |
| Cooking Fuel | 0·1543 | 0·0580 | 0·2518 | -0·0025 | 0·0156 | -0·1753 | -0·4019 | 0·6125 | 0·1315 | -0·0197 | -0·0054 | 0·0039 | 0.0058 | 0.0227 | 1.0000 |  |  |
| OCP use | -0·0324 | -0·0185 | 0·0320 | -0·0059 | 0·0155 | 0·0505 | 0·0253 | -0·0161 | 0·0211 | -0·0103 | 0 ·0054 | -0 ·0160 | -0.0135 | -0.0078 | -0.0110 | 1.0000 |  |
| BMI | -0·1777 | -0·0223 | -0·1295 | 0·0494 | -0·0332 | 01359 | 0·2503 | -0·3613 | -0·0691 | -0·0152 | 0·0129 | -0·0040 | 0.0001 | 0.0249 | -0.2588 | -0.0013 | 1.0000 |
| Note- only highlighted values selected for the interaction test due to strong correlation between them | | | | | | | | | | | | | | | | | |
