# Supplementary material for: Unhealthy behaviours associated with uncontrolled hypertension among adults in India- Insights from a national survey
Source: PLoS One. 2025 Jan 17;20(1):e0310099. doi: 10.1371/journal.pone.0310099 (PMC11741589; doi:10.1371/journal.pone.0310099)
Supplement: S4 Table — *Estimated values are adjusted with age groups, marital status, current working status, religion, ethnicity, tobacco use, alcohol use, dietary diversity, covered health insurance, diabetic, heart disease, OCP use, and BMI. (DOCX) [file pone.0310099.s004.docx]

| **S4 Table b2: Adjusted effect of uncontrolled hypertension among females with interaction results** | | | |
| --- | --- | --- | --- |
| **Wealth Index vs Education** |  |  |  |
| Poorest vs higher (ref) | Odds ratio | P-value | 95% CI |
| Poorer vs primary | 0**·**43 | 0**·**071 | 0**·**17-1 **·**08 |
| Poorer vs secondary | 0 **·**67 | 0**·**360 | 0**·**28-1**·**58 |
| Poorer vs higher | 1**·**01 | 0 **·**994 | 0**·**15-6 **·**76 |
| Middle vs Primary | 0 **·**62 | 0**·**329 | 0**·**24-1**·**61 |
| Middle vs secondary | 0**·**79 | 0**·**588 | 0**·**34-1 **·**86 |
| Middle vs higher | 1**·**34 | 0**·**660 | 0**·**36-4**·**99 |
| Richer vs primary | 1**·**13 | 0**·**816 | 0**·**42-3**·**04 |
| Richer vs secondary | 0**·**61 | 0**·**274 | 0**·**25-1**·**48 |
| Richer vs higher | 1**·**18 | 0**·**781 | 0**·**37-3**·**77 |
| Richest vs primary | 0**·**44 | 0**·**137 | 0**·**15-1**·**30 |
| Richest vs secondary | 0**·**41 | 0**·**068 | 0**·**16-1**·**07 |
| Richest vs higher | 1 (omitted) |  |  |
| **Wealth Index vs Place of Residence** |  |  |  |
| Poorest vs urban (ref) |  |  |  |
| Poorer vs rural | 1**·**41 | 0**·**681 | 0**·**28-7**·**17 |
| Middle vs rural | 0**·**98 | 0 **·**977 | 0**·**21-4**·**54 |
| Richer vs rural | 1**·**32 | 0 **·**718 | 0 **·**29-5 **·**98 |
| Richest vs rural | 1**·**40 | 0**·**659 | 0**·**31-6**·**35 |
| **Cooking Fuel vs Wealth Index** |  |  |  |
| Polluting vs Poorest (ref) |  |  |  |
| Clean vs poorer | 4 **·**93 | 0**·**020 | 1**·**29-18**·**80 |
| Clean vs middle | 6**·**91 | 0**·**005 | 1**·**80-26**·**50 |
| Clean vs richer | 5 **·**24 | 0**·**018 | 1**·**33-20**·**74 |
| Clean vs richest | 4**·**46 | 0**·**065 | 0**·**91-21**·**78 |
| **Cooking fuel vs Residence** |  |  |  |
| Polluting vs Urban (ref) |  |  |  |
| Clean vs rural | 1**·**27 | 0**·**524 | 0**·**61-2**·**66 |
| *Estimated values are adjusted with age groups, marital status, current working status, religion, ethnicity, tobacco use, alcohol use, dietary diversity, covered health insurance, diabetic, heart disease, OCP use, and BMI | | | |
